# Impact of free summer day camp on physical activity behaviors and screentime of elementary-age children from low-income households: a randomized clinical trial

**DOI:** 10.1186/s12966-025-01852-2

**Published:** 2025-12-29

**Authors:** Michael W. Beets, Sarah Burkart, Christopher D. Pfledderer, Elizabeth Adams, R. Glenn Weaver, Bridget Armstrong, Keith Brazendale, Xuanxuan Zhu, Brian Chen, Alexander McLain

**Affiliations:** 1 Arnold School of Public Health, University of South Carolina, Columbia, SC USA; 2 UTHealth Houston, School of Public Health, Houston, USA; 3 Department of Health Sciences, University of Central Florida, Orlando, USA

## Abstract

**Background:**

To examine the efficacy of providing free summer day camp (SDC) to children from low-income families on changes in physical activity, time spent sedentary, and screentime.

**Methods:**

Across three summers (2021–2023), we randomized 422 children (8.2 ± 1.5yrs, 48% female, 51% Black, 69% at or below 200% Federal Poverty Level, 30% food insecure) from seven elementary schools to one of two conditions: summer as usual (control, *n* = 199) or free SDC for 8-10wks (intervention, *n* = 223). Accelerometry measured activity (moderate-to-vigorous PA [MVPA] and time spent sedentary) and parent daily report of screentime were measured using a 14-day in April/May (school) and July (summer). Intent-to-treat analysis examined changes in behaviors between school and summer. Exposure models examined differences in behaviors during summer on days when children attended vs. did not attend a SDC in both intervention and control children.

**Results:**

Intent-to-treat models indicated in the summer children in the intervention group accumulated + 15.0 min/day (95CI 12.0 to 18.0) more MVPA and spent − 29.7 min/day (-37.7 to -21.8) less time sedentary and − 14.1 min/day (-23.9 to -4.3) on screens, compared to children in the control group. Exposure models indicated, on days children attended SDCs, they accumulated more MVPA (+ 26.1 min/day, 22.5 to 29.7), and spent less time sedentary (-63.5 min/day, -72.9 to -54.1) and on screens (-9.5 min/day, -20.1 to 1.2), compared to days when children did not attend SDC.

**Conclusions:**

Policies targeting upstream structural factors, such as universal access to existing community SDCs during summer, could lead to improvements in health behaviors among children from low-income households.

**Clinical trials.Gov:**

NCT04072549

**Supplementary Information:**

The online version contains supplementary material available at 10.1186/s12966-025-01852-2.

## Introduction

In the United States, in addition to school-based interventions, summer vacation has emerged as a key time to address unhealthy weight gain among youth. Extensive evidence indicates youth gain more weight relative to height during the 3-months of summer (June, July, and August) compared to the entire 9-month school year (September through May) [1–21]. One of the leading hypotheses to understand this phenomenon is the Structured Days Hypothesis [22, 23]. The Structured Days Hypothesis posits youth engage in less health promoting behaviors during summer, compared to the school year [22, 24]. The hypothesized reason for such changes in health behaviors is due to the limited time youth spend in a structured setting during summer. When school is in session, attending school represents a consistent (5 days per week, ~7 h per day, ~ 180 days per year) structured setting with pre-planned, segmented, and adult-supervised compulsory environments [22, 23, 25, 26]. Through environmental control and limit setting, youth health behaviors are beneficially modified. Potential behavioral drivers of unhealthy weight gain during summer include the time youth spend physically active and the amount of time they spend on screens (e.g., TV, handheld devices) [27, 28]. Observational studies indicate sedentary behaviors increase, while time spent in moderate-to-vigorous physical activity (MVPA) declines during summer [22, 29–31]. Intervening upon these movement behaviors is challenging, especially during the summer where children do not regularly convene in large numbers, like when they are in school, to deliver a behavioral intervention.

During the summer, fewer universally accessible structured environments are available, which leads to increased discretionary, unstructured time, and with this, a decline in health promoting behaviors.

With more than 20,000 operating in the US, serving over 12 million youth, summer day camps represent a key setting over the summer to promote physical activity and reduce screen time [22, 25, 32, 33]. Summer day camps (SDC) exhibit hallmark characteristics of school, they are pre-planned, segmented, and adult supervised compulsory environment that last + 7 h per day throughout most of the summer [34]. Observational studies indicate youth accumulate a substantial amount of MVPA while attending SDCs [34, 35]. However, findings from several pilot/feasibility studies examining the impact of sending youth to SDCs on MVPA and screen time are mixed [9, 10, 36–38]. Several studies indicate sending youth to SDCs does not improve the time spent in MVPA or sedentary or on screens. However, analyses comparing days when youth attended a SDC compared to days when they did not, or the more days a child attends a SDC is associated with greater MVPA and less time sedentary or on screens indicating that days with structure can aid in maintaining or improving healthy behaviors in children [36, 39]. 

Summer day camps in the US largely operate on a fee-for-service model, with parents paying out-of-pocket for their children to attend. Typical cost for one week at a summer day camp range from $75 to over $400 [32, 40]. For families from low-income households, attending SDCs may be financially impracticable, even if they would like their child to attend, thereby creating inequities in accessibility of a potentially health benefiting setting during the summer [26, 32, 41]. Studies show youth from low-income households are more at-risk for unhealthy weight gain during the summer and these same youth are less likely to be enrolled in summer programs [19, 20, 32]. This implies they would also have more unfavorable activity and sedentary behavior during summer.

The current study presents secondary health behavior outcomes from an efficacy trial on the impact of sending children from low-income households to SDC for free [42]. The primary outcome of this randomized trial was change in Body Mass Index z-scores during the summer. The study demonstrated children who received free SDC reduced their zBMI during the summer compared to children randomized to summer as usual [42]. Consistent with the Structured Days Hypothesis, we hypothesized children randomized to receive free SDC would exhibit greater minutes of MVPA, and lower minutes of sedentary and screen behaviors compared to children experiencing summer as usual.

## Methods

This randomized controlled trial was conducted during summer 2021, 2022, and 2023. The study followed the Consolidated Standards of Reporting Trials (CONSORT) reporting guidelines. All procedures were approved by the Institutional Review Board of the University of South Carolina and the study was preregistered at Clinicaltrials.gov (NCT04072549). This study presents the evaluation for the secondary outcomes of accelerometry-derived MVPA and time spent sedentary, and parent-report of screen time use between intervention and control children.

### Study design and participants

We partnered with seven Title I elementary schools from a single school district in a mid-sized metropolitan city in the southeastern United States that predominantly serves children from low-income families. In the spring (March-April) of each school year, we sent invitations to parents of children enrolled in kindergarten through 4th grade via each school’s social worker, paper flyers, and electronic communications (i.e., online newsletter and Class Dojo). Children were eligible to participate if they were enrolled in one of our partner Title I schools, did not anticipate moving during the summer, and were able to complete the measures during the spring and summer (e.g., not out-of-state visiting relatives during the entire summer). Informed consent was obtained from one parent and/or guardian through an online Health Insurance Portability and Accountability Act (HIPAA)-compliant web-based survey platform accessed via a smartphone. Across the study timeline, a total of three cohorts of children were recruited and randomized to receive the intervention or the control group. This allowed for the distribution of the cost to send children to summer day camp across multiple years of the overall study’s budget.

### Randomization and blinding

Once parents completed the informed consent, which confirmed their participation in the study, children were randomly allocated to either control (i.e., summer as usual) or intervention (i.e., free SDC), stratified by number of assistance services used/received (0–3 vs. 3 or more; i.e., Welfare, Temporary Assistance for Needy Families (TANF), Temporary Cash Assistance (TCA), Children’s Health Insurance Program (CHIP), Medicaid, Supplemental Nutrition Assistance Program (SNAP), Women, Infants and Children (WIC), or Supplemental Security Income (SSI)) within family units, grade in school, and child gender. Where parents had two or more eligible children (average of 1.3 siblings per household) siblings were grouped together to ensure siblings were randomized to the same condition. Children were allowed to participate in the study for a single summer. Randomization occurred prior to baseline assessments each May. The decision to randomize at this time was made to treat the participating families with dignity and be respectful of their needs in making alternative summer care arrangements if their child was not randomized to receive free SDC. Randomization was performed using a stratified random number generator in Stata (v17, StataCorp LLC. College Station, Texas) by a co-investigator (R.G.W.) external to the day-to-day conduct of the trial. The lead investigator (M.W.B.) and data collectors were blinded to allocation.

### Intervention and control conditions

The intervention group was assigned to receive 8 to 10 weeks of free SDC to provide programming for the entire summer vacation period. The difference in the number of weeks was due to summer 2021 being shorter than summers 2022 and 2023 because of COVID-19 school closures and subsequent calendar adjustments. The SDC that served as the intervention was operated by a local parks and recreation commission, which operated multiple summer programs across the school district. The SDC provided indoor and outdoor opportunities for children to be physically active each day, included enrichment and academic programming, weekly field trips, and provided breakfast, lunch, and snacks. The foods served adhered to existing federal food program nutrition guidelines by the USDA and were reimbursed via the Summer Food Service Program (https://www.fns.usda.gov/summer/sunmeals). The SDC maintained a ratio of 1 staff member for every 12 children enrolled – consistent with South Carolina childcare regulations. The SDC operated daily (Mon-Fri) for 8 to 10 weeks during the summer (except for the 4th of July holiday week). The SDC opened at 7am for morning drop-off and ended at 5pm each day. Based on the schedules of the SDC, physical activity opportunities were scheduled for 3 to 4 h each day, with the remaining 4 to 5 h dedicated to enrichment/academic content or field trips (example schedules can be found in the Supplementary material 1). The local parks and recreation commission had a history of operating SDCs in the community and had the appropriate safety and staff training certifications to ensure a safe/nurturing environment. Each summer children randomized to receive free SDC attended the same SDC operated by the same organization. The SDC operated according to routine practice, with no outside assistance or modifications from the investigative team. In summer 2022 and 2023, the school district provided transportation for children assigned to the intervention group. No transportation was provided in 2021 due to COVID-19. The control group did not receive free SDC programming and only children randomized to receive free summer day camp were allowed to attend the SDC offered by our community partner. These children experienced what we consider “summer as usual” (see Structured Programming Exposure below).

#### Measures

### Physical activity and sedentary behaviors

Physical activity (PA) and time spent sedentary were measured using a wrist-placed accelerometry (ActiGraph GT9X) on the non-dominant wrist with a 24-hours wear protocol for 14-days [43, 44]. The ActiGraph GT9X accelerometer is a triaxial research-grade accelerometer frequently used in studies measuring children’s free-living 24-hour behaviors (i.e., PA, sedentary behavior, sleep). ActiGraph GT9X accelerometers were initialized and downloaded using Actilife software (version 6.13.4, ActiGraph LLC). Accelerometers were initialized to record data at a frequency of 30 Hz. Stop time was not used. Idle sleep mode was enabled to preserve battery life and the display was turned off to limit distractions for children while attending school.

Assessment of PA behaviors were collected at two timepoints each year – during school (April/May, average daylight 13 h) and again during summer (mid-July, average daylight 14 h). Children were instructed to wear the device during school and summer for 14 consecutive days during each assessment period. Devices were distributed and returned by mail. Each mailing contained a device and information regarding wear procedures (e.g., wear while awake and sleeping, waterproof). Data were downloaded and saved in raw format as.gt3x files and were processed using the GGIR package (version 2.8-2) in R (Version 4.1.2; R Foundation for Statistical Computing; Vienna, Austria). Time spent in PA intensity categories was determined using intensity thresholds described by Hildebrand et al. [45, 46]. A valid wear day was defined as a minimum of 16 h and participants were included if they had at least one day of data [47]. The primary metrics of interest were time spent in MPVA and time spent sedentary in minutes per day.

### Screen time

Each evening of the 14-day accelerometer protocol, parents received a text via their smartphone to complete a time use record (see “*Structured Program Exposure – Time Use Record*” section below for details). One of the questions of the time use record asked parents to estimate the total amount of time (hours and minutes) their child spent in front of a screen that day (e.g., TV, computer, video game, smartphone, and tablet).

### Child and parent demographics

Each spring (April/May) parents completed a brief survey via their smartphone to collect information about the child (e.g., biological sex, parent self-reported race/ethnicity), household income, parent highest education level obtained, household food insecurity status, and the use/receipt of any of the following public assistance services: Welfare, Temporary Assistance for Needy Families (TANF), Temporary Cash Assistance (TCA), Children’s Health Insurance Program (CHIP), Medicaid, Supplemental Nutrition Assistance Program (SNAP), Women, Infants and Children (WIC), or Supplemental Security Income (SSI). Participants were compensated with a $25 gift card for completing the brief survey.

### Structured program exposure – time use record

We recognized that children randomized to the control condition (i.e., summer as usual) could voluntarily elect to enroll in some type of summer programming. Based on national data, we anticipated approximately 20% of the children randomized to the control would attend some type of summer programming [48]. Conversely, we recognized children randomized to receive free SDC could elect to not attend the free SDC or attend some other summer program offered. To account for differential exposure to structured programing during the summer in both the control and intervention groups, we collected information regarding structured programing attendance using an electronic time use record (TUR).

The TUR was developed in Qualtrics and distributed to parents at 8PM each evening of the 14-day accelerometer wear protocol during school and summer to complete on their smartphone. The TUR was based on the day reconstruction method and was designed to be minimally intrusive and maximally representative of one’s day [49]. The time use record involved parents completing a series of questions about their child’s previous 24 h. The first question asked about the time their child went to bed the previous night, followed by when their child woke today. The next series of questions asked about whether their child left where they were (e.g., woke up at home, did child leave home today?) and if “yes”, where their child went and at what time (e.g., left home and went to school at 7:15AM). Parents reported time in 15 min increments (e.g., 9AM, 9:15AM, 9:30AM…). This sequence of questions continued until parents reported the entire day with where their child was and at what times. The TUR provided a list of common locations where a child may be that a parent could select from: home, before school program, school, afterschool program, sports, music/theater/dance, neighborhood pool/water, park/river/lake/beach, park/playground, employment/volunteer, church/synagogue/mosque or other religious activity, friend’s house, relative’s house, restaurant/fast food outlet, errands/shopping/appointments, tagging along/attending family or friend’s event/activity, or summer day camp. There was an additional “other” option for parents to write a specific activity/setting not represented. For the purpose of this study, we classified before school programs, afterschool programs, sports, music/theater/dance, employment/volunteer, and summer day camps as structured settings. For our secondary analyses examining exposure to structure (see below), we excluded school as a structured setting given this was universal for all children when school was in session, and thus, estimates of the impact of structure during school (April/May) represent the amount of structure above and beyond the school day in the hours after school ended. Each TUR took ~ 7 min to complete. Completed TUR were matched by date with valid accelerometer days and used to estimate exposure to structured programming during school and summer in both groups.

### Statistical power

Sample size calculations for this randomized controlled trial were originally based on the ability to detect a statistically significant group (intervention vs. control) by time (before summer to end of summer) difference in the change in the primary outcome of zBMI (Cohen’s *d* = 0.2) with a two-group mixed-effects model with statistical power of 0.80 and an alpha of 0.05. Accounting for the intraclass correlation of 0.06 at the school level [50] with a variance inflation factor of 2.16 (average recruitment of 20 children per school across 6 schools) and an anticipated attrition rate of 25%, the total number of children required to be enrolled at baseline was 420 to arrive at a final sample of 330 (165 per condition). Calculations were performed using G*power (v.3.1.9.3) and Microsoft Excel.

### Statistical analyses

The primary analysis was intent-to-treat (ITT), defined as using all available data from children according to the original group they were randomized (free summer day camp or summer as usual) regardless of their attendance at a summer program (e.g., intervention children never attending the SDC and control children attending some other form of SDC). Analyses used mixed-effects models, accounting for the nesting of repeated measures within children, families, and schools. The treatment effect of interest was the group (intervention vs. control) and time (pre summer vs. post summer) interaction. Missing data on the dependent variables of interest of MVPA, time spent sedentary, and screen time were accounted for using full information maximum likelihood estimation [51], allowing for all children’s data to be used regardless of missing data at either pre or post summer. Covariates included in the model were the child’s biological sex [52] and age [53], self-reported race/ethnicity [54], parent education [55], food insecurity status [56], and poverty level [57]. Missing data on covariates were handled using multivariate multiple imputation with the covariates serving as variables to inform the imputation of 100 datasets for both continuous and categorical covariates using STATA v18.0 multiple imputation commands.

Secondary analyses (i.e., exposure models) examined the exposure to structured programing and included both the intervention and control groups. For these analyses we defined exposure to structure as any day where a child is reported on the TUR to have attended a structured setting outside the home (attended/not attend, binary variable). This choice of dichotomizing structured settings at the day level was done because the distribution of minutes in a structured setting was heavily skewed in the summer. Children (both intervention and control), when they were reported to have attended a structured setting in the summer, did so for a day-long program (i.e., 7–8 h), which for the intervention group was the typical length of attending the SDC. This means that for the children in the control group, when they did attend structured programming in the summer, this programming lasted for approximately the same length of time as the intervention group. Because all children were exposed to school, exposure to structured programming during school (April/May) excluded attending school and, therefore, represents only additional structured programming a child was exposed to above and beyond the school day. In addition to changes in the continuous outcomes, we examined the probability of meeting MVPA (≥ 60 min per day) and screen time (≤ 120 min per day) guidelines [58] using both the above stated ITT and exposure to structured programming models. All models included both weekdays and weekend days. Because free SDC was provided on weekdays only, we also conducted the same ITT and exposure analysis restricting the data to only weekdays. The assumptions for all models (normality of residuals, homoscedasticity) were checked using; no violations were found. All models were conducted using STATA v18.0 (College Station, Texas).

## Results

**Fig. 1:**
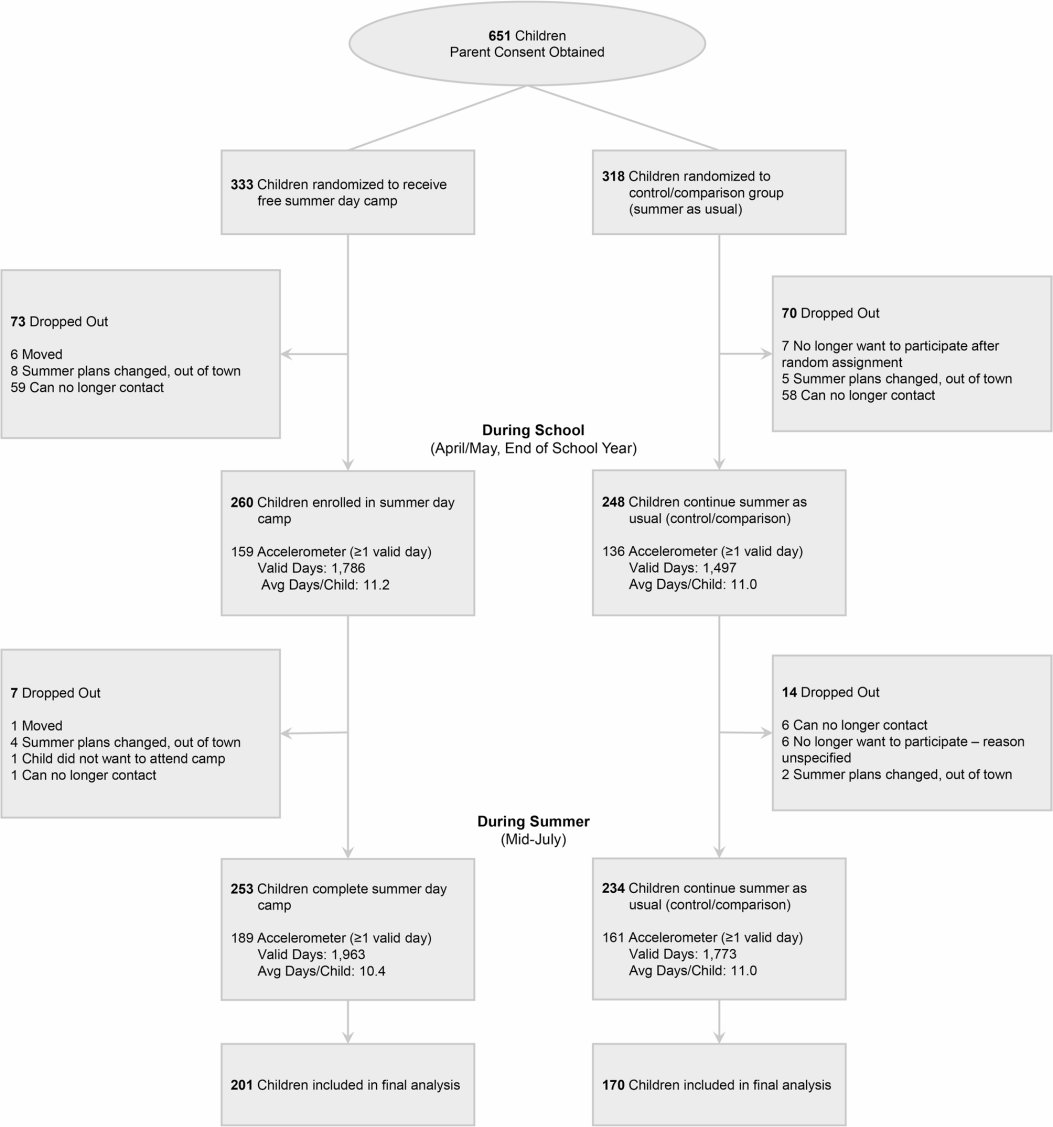
CONSORT Diagram

Of the 651 parents who expressed interest in participating in the study and completed the online informed consent, we were able to recontact 525 to inform them of their random assignment (Fig. 1). This resulted in 260 children randomized to receive free summer day camp and 248 children randomized to continue summer as usual. Of these, 215 intervention and 181 control children provided 7,019 days (avg. 20.1 ± 7.0 days per child) for analyses across baseline (avg. 10 days per child for end of school year April/May, prior to summer) and summer (avg. 9.6 days per child during summer, mid-July) timepoints. During the school measurement period, 87% of children had 4 or more valid days of accelerometry which comprised 98% of valid days, while during the summer 81% of children had 4 or more valid days of accelerometry which comprised 97% of valid days. The demographics of children who provided accelerometer data are presented in Table 1. Supplemental Table 1 compares children who provided valid accelerometer data with those that did not provide accelerometer data by summer and school time periods by intervention and control groups. During school on weekdays, parents of intervention and control children indicated their child spent 30% and 28%, respectively, of their weekdays in a structured program outside of school (Table 1). During summer on weekdays, parents of children randomized to the control reported children spent a 29% of their days in a structured program compared to 75% of days spent in a structured program for children randomized to the intervention (Table 1).

**Table 1:** Baseline demographics of children with accelerometer data during school and during summer for the intervention (i.e., free summer day camp) and control (i.e., summer as usual) groups

	SCHOOL			SUMMER		
	Control (Summer As Usual)	Intervention (Free Summer Day Camp)	P-Value	Control (Summer As Usual)	Intervention (Free Summer Day Camp)	P-Value
Sample Size	136	159			189	0.700
Child Characteristics						
Sex (Female, %)	49%	42%	0.278	49%	47%	0.700
Age (years, mean, ±SD)	8.2 ± 1.6	8.2 ± 1.6	0.715	8.2 ± 1.6	8.4 ± 1.5	0.240
Self-Identified Race/Ethnicity, %			0.230			0.921
Hispanic	8%	11%		6%	10%	
Black	42%	43%		45%	44%	
White	37%	39%		39%	36%	
Other Race	3%	6%		2%	8%	
Multiple Races	10%	1%		8%	1%	
Anthropometrics (baseline)						
BMI Z-Score (Median, IQR)	0.74 (−0.1, 1.6)	0.75 (0.0, 1.8)	0.894	0.76 (−0.2, 1.5)	0.79 (−0.0, 1.8)	0.842
BMI classifications (%)			0.393			0.528
Normal Weight	60%	63%		63%	64%	
Overweight	22%	16%		18%	14%	
Obese	18%	21%		18%	22%	
Household/Family (%)						
Food Insecure	31%	27%	0.473	37%	25%	0.020
At or Below 200% Federal Poverty Level	62%	65%	0.622	60%	62%	0.565
Services Received (%)						
Welfare, Temporary Assistance for Needy Families (TANF), or Temporary Cash Assistance (TCA)	20%	16%	0.326	22%	13%	0.041
Children’s Health Insurance Program (CHIP), Medicaid	52%	51%	0.838	49%	49%	0.934
Supplemental Nutrition Assistance Program (SNAP)	17%	26%	0.066	25%	25%	0.920
Women, Infants and Children (WIC)	9%	6%	0.444	8%	12%	0.189
Parent Education (%)			0.059			0.337
Some college or less	41%	51%		44%	50%	
2 to 4 year degree	35%	34%		36%	36%	
Graduate degree	24%	14%		21%	15%	
Family Income (%)			0.188			0.104
$30k or less	39%	49%		38%	49%	
$30k to $60k	32%	23%		29%	21%	
$60k or more	29%	28%		33%		
Structured Setting Attendance						
Percentage of Weekdays	28%	30%	0.284	29%	75%	0.001
Average Minutes Spent in Structured Setting (mean, ±SD)	38.3 ± 73.6	38.0 ± 68.4	0.937	106.9 ± 205.5	354.4 ± 249.5	0.001
Average Minutes Spent in Structured Setting for Days with Structure) mean, ±SD) (does not include days with “zero” minutes)	144.3 ± 71.5	137.0 ± 57.6	0.005	416.8 ± 188.5	502.5 ± 117.3	0.001

### Intent-to-treat (ITT) analyses

**Fig. 2:**
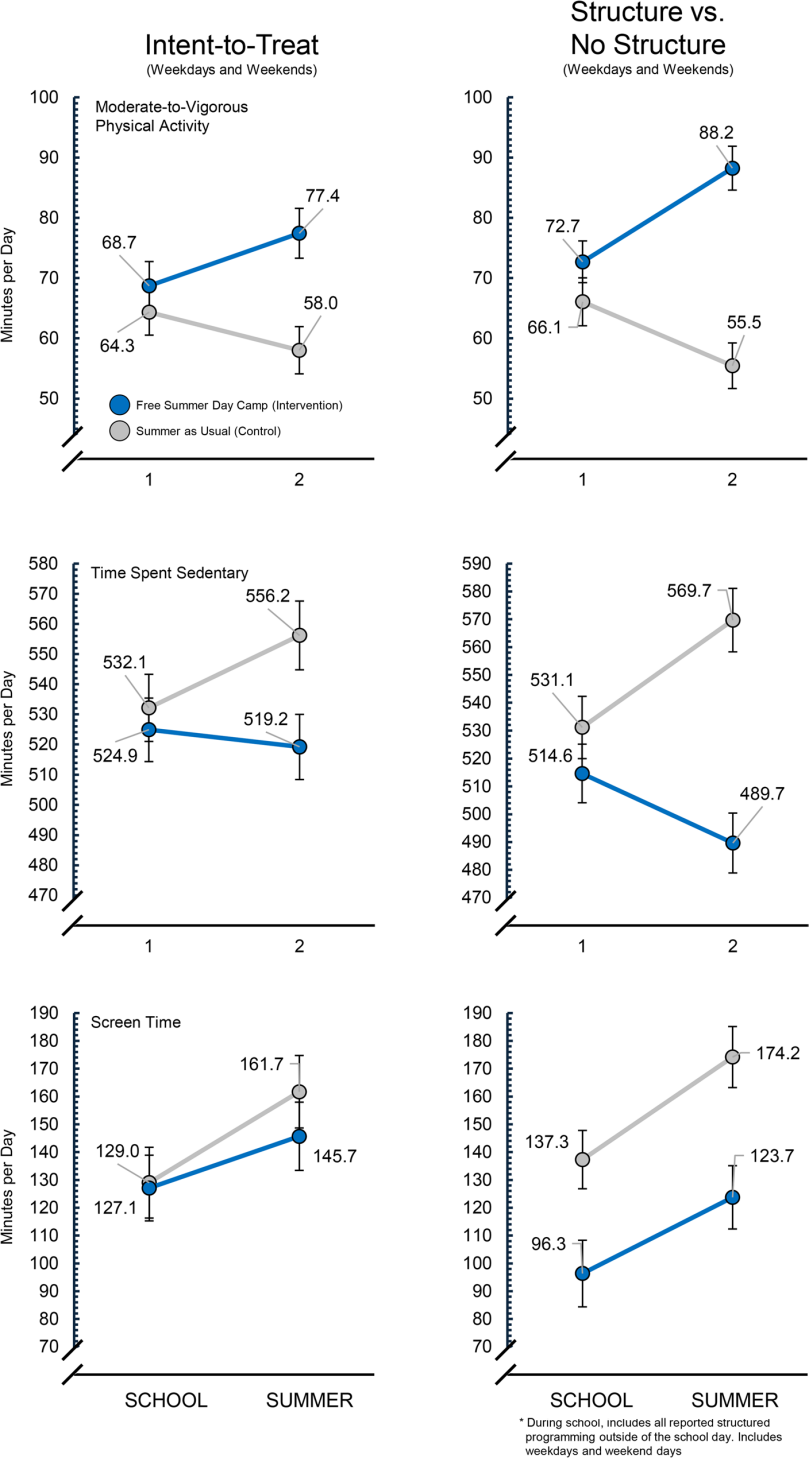
Intent-to-Treat and exposure to structure between group differences in moderate-to-vigorous physical activity, time spent sedentary, and screentime use between Intervention (*n* = 223) and Control (*n* = 199) groups based on adjusted model estimates and including weekdays plus weekend days

The group-x-time interaction (i.e., difference-in-difference) effect for MVPA, time spent sedentary, and screen use were statistically significant (see Table 2; Fig. 2) favoring children receiving free summer day camp (i.e., intervention group). Specifically, MVPA increased in the intervention group by + 15.0 min per day during summer compared to the control group, whereas the intervention group decreased time spent sedentary by −29.7 min per day and time using screens by −14.1 min per day during the summer compared to the control group. The ITT analyses restricted to only weekdays showed a greater effect, with MVPA increasing by + 23.6 min per day, while time spent sedentary and using screens decreased by −46.2 and − 25.0 min per day, respectively, for the intervention group compared to the control group during summer (see Table 2; Fig. 2).

**Table 2:** Between group differences in change in Moderate-to-Vigorous physical activity (MVPA), time spent Sedentary, and screen use (minutes per day) and the probability (Odds Ratio) of meeting MVPA and screen use guidelines

				School			Summer				Contrast of Interest	Minutes per Day			Meeting Guidelines		
Outcome	Model	Days of Week	Condition	M	±SE		M	±SE		Δ		Est	(95CI)		OR	(95CI)	
Moderate-to-Vigorous Physical Activity	Intent-to-Treat	Weekdays + Weekends	Control	64.3	± 2.1		58.0	± 2.1		−6.3	Difference in **Δ** (school to summer) between Conditions	**15.0**	**(12.0**,	**18.0)**	**2.98**	**(2.35**,	**3.79)**
			Intervention	68.7	± 1.9		77.4	± 2.0		8.7							
			Δ	4.4			19.4			15.0							
		Weekdays only	Control	65.8	± 2.1		68.4	± 2.0		2.6	Difference in **Δ** (school to summer) between Conditions	**23.6**	**(19.9**,	**27.3)**	**4.98**	**(3.54**,	**6.99)**
			Intervention	60.2	± 2.2		86.3	± 2.0		26.2							
			Δ	−5.6			18.0			23.6							
	Exposure	Weekdays + Weekends	No Structure	66.1	± 1.8	±	55.5	± 1.9		−10.6	Difference in **Δ** (school to summer) between Conditions	**26.1**	**(22.5**,	**29.7)**	**6.32**	**(4.54**,	**8.80)**
			Structure	72.7	± 2.0		88.2	± 1.9		15.5	Difference between Structure & No Structure at School	**6.6**	**(3.9**,	**9.4)**	**1.99**	**(1.57**,	**2.51)**
			Δ	6.6			32.8			26.1							
		Weekdays only	No Structure	65.5	± 1.6		56.2	± 1.8		−9.2	Difference in **Δ** (school to summer) between Conditions	**26.3**	**(22.3**,	**30.2)**	**8.29**	**(5.59**,	**12.30)**
			Structure	73.2	± 1.8		90.3	± 1.7		17.1	Difference between Structure & No Structure at School	**7.7**	**(4.7**,	**10.7)**	**1.90**	**(1.44**,	**2.51)**
			Δ	7.7			34.0			26.3							
Time Spent Sedentary	Intent-to-Treat	Weekdays + Weekends	Control	532.1	± 5.7		556.2	± 5.8	532.1	± 5.7	Difference in **Δ** (school to summer) between Conditions	**−29.7**	**(−37.7**,	**−21.8)**			
			Intervention	524.9	± 5.4		519.2	± 5.5	524.9	± 5.4							
			Δ	−7.2			−37.0		−7.2								
		Weekdays only	Control	533.1	± 5.5		521.8	± 5.1		−11.3	Difference in **Δ** (school to summer) between Conditions	**−46.2**	**(−56.0**,	**−36.5)**			
			Intervention	562.8	± 5.7		505.2	± 5.4		−57.5							
			Δ	29.7			−16.5			−46.2							
	Exposure	Weekdays + Weekends	No Structure	531.1	± 4.5		569.7	± 4.7		38.6	Difference in **Δ** (school to summer) between Conditions	**−63.5**	**(−72.9**,	**−54.1)**			
			Structure	514.6	± 5.2		489.7	± 4.9		−25.0	Difference between Structure & No Structure at School	**−16.5**	**(−23.7**,	**−9.4)**			
			Δ	−16.5			−80.0			−63.5							
		Weekdays only	No Structure	529.8	± 3.5		579.0	± 4.0		49.2	Difference in **Δ** (school to summer) between Conditions	**−76.6**	**(−86.8**,	**−66.4)**			
			Structure	515.1	± 4.3		487.6	± 3.9		−27.5	Difference between Structure & No Structure at School	**−14.7**	**(−22.3**,	**−7.1)**			
			Δ	−14.7			−91.3			−76.6							
Screen Time	Intent-to-Treat	Weekdays + Weekends	Control	129.0	± 6.5		161.7	± 6.6		32.7	Difference in **Δ** (school to summer) between Conditions	**−14.1**	**(−23.9**,	**−4.3)**	1.21	(0.90,	1.63)
			Intervention	127.1	± 6.0		145.7	± 6.3		18.6							
			Δ	−1.9			−16.0			−14.1							
		Weekdays only	Control	115.7	± 7.4		113.4	± 6.9		−2.3	Difference in **Δ** (school to summer) between Conditions	**−25.0**	**(−35.5**,	**−14.6)**	**1.61**	**(1.11**,	**2.31)**
			Intervention	164.4	± 7.5		137.1	± 7.1		−27.3							
			Δ	48.7			23.7			−25.0							
	Exposure	Weekdays + Weekends	No Structure	137.3	± 5.3		174.2	± 5.6		36.9	Difference in **Δ** (school to summer) between Conditions	−9.5	(−20.1,	1.2)	0.96	(0.68,	1.35)
			Structure	96.3	± 6.1		123.7	± 5.8		27.4	Difference between Structure & No Structure at School	**−41.0**	**(−49.0**,	**−32.9)**	**3.19**	**(2.45**,	**4.16)**
			Δ	−41.0			−50.4			−9.5							
		Weekdays only	No Structure	122.9	± 5.7		178.3	± 6.1		55.5	Difference in **Δ** (school to summer) between Conditions	**−25.0**	**(−36.3**,	**−13.6)**	1.12	(0.74,	1.71)
			Structure	94.4	± 6.3		124.9	± 6.0		30.5	Difference between Structure & No Structure at School	**−28.4**	**(−37.0**,	**−19.8)**	**2.64**	**(1.92**,	**3.64)**
			Δ	−28.4			−53.4			−25.0							

BOLDED PINK values are statistically significant with 95% Confidence Intervals that do not include zero

Exposure = Days attending structured programming (parent report from time use record) vs. days without structured programming

### Exposure to structured programming analyses

The analyses examining the differences in MVPA, time spent sedentary and using screens when attending structured programming and not attending structure programming are presented in Table 2; Fig. 2. Structured programming had a beneficial impact on all three behaviors during both school and summer. Specifically, structured programming during school (considering week and weekend days) was associated with a + 6.6, −16.5, and − 41.0 min per day of MVPA, sedentary, and screen use, respectively, compared to days where children did not attend structured programming. The structure-x-time interaction for summer indicated structured programming was associated with a + 26.1, −63.5, −9.5 min per day of minutes per day of MVPA, sedentary, and screen use, respectively, compared to days where children did not attend structured programming. For weekdays only, increases in MVPA and decreases in time spent sedentary were largest (see Table 2).

### Odds of meeting MVPA and screen time guidelines

The ITT analysis indicated the odds of meeting the MVPA 60 min per day guideline increased by 2.98 (weekday + weekend) and 4.98 (weekday only) (see Table 2). The odds of meeting 120 min or fewer of screen use per day guideline showed a non-statistically significant ITT effect considering week plus weekend days (OR 1.20) whereas a statistically significant effect was observed considering weekdays only (OR 1.60). The exposure to structured programming models showed days attending structured programming was associated with an increased odds of meeting the MVPA guideline during school and summer, with an increase in the odds occurring during summer (group-x-time interaction), whereas for screen time there was an overall increase in the odds of meeting the screen time guideline, during both school and summer, but there were no statistically significant time-group interactions for summer (see Table 2).

## Discussion

This randomized controlled trial evaluated the efficacy of providing free access to SDCs for children predominantly in households with low-income on their physical activity and sedentary behaviors. Compared to children randomized to experience summer as usual, those children who received free access to a SDC accumulated substantially greater amounts of MVPA and reduced the amount of time they spent sedentary during summer. Findings for screen use showed exposure to structure reduced screen time equally during the school year and summer. These findings have important implications for leveraging structured settings to address unhealthy behavior change, and potentially excessive weight gain, during the summer.

Our findings demonstrate accessing readily available and already operating SDCs within a community can serve as a viable solution to address the decline in health behaviors observed during summer. A key component to emphasize of our study is that we deliberately elected not to alter the routine practice within the SDCs, but rather let the SDCs operate as usual. Doing so provided a real-world demonstration of the impact of routine practice within SDCs on children’s health behaviors. This has major implications for designing effective interventions, as typical setting-based approaches to improve the health behaviors of children involve extensive staff training and oversight, purchasing of pre-packaged health promotion curricula, or the reliance upon outside experts. While such approaches can result in improvements in health behaviors, these have limited sustainability [59], nor have they resulted in the sizable improvements demonstrated by our study [60, 61]. Specifically, complex behavioral interventions relying upon electronic monitoring devices on TVs or interventions with TV viewing contingent upon exercise reduce screen viewing − 9 to −171min/day [61]. Setting-based interventions that rely upon a variety of child- and setting-focused intervention components improve MVPA an additional 2 to 4 min/day [60, 62]. In contrast, sending children to free SDC reduces screen time − 14.1 min/day (exposure model − 40.9 min/day) while increasing MVPA by + 15.0 min/day (exposure model + 26.1 min/day). Our findings provide evidence for upstream policy changes whereby efforts to support equitable access to summer programming, such as using tax subsidies, could mitigate the opportunity disparities during the summer and lead to improved health behaviors of children from low-income households. In the US, providing financial assistance to cover some or all of the costs associated with attending structured programming, namely in the form of public K-12 education and Head Start childcare, is widespread. Our findings indicate that if financial support is extended to summer can lead to a reduction in engaging in unhealthy behaviors.

These changes show the considerable influence a setting, such as SDCs, can have upon the health behaviors of children. This is particularly relevant given SDCs are one of the more universal settings during the summer for children to attend, yet for many children from low-income households, access is limited due to financial barriers [32, 41, 63]. Simply providing access, without extensive changes to routine operation, can lead to important and sizable changes in health behaviors such as improved physical activity, reduced time spent sedentary and on screens. Our findings suggest providing universal access to summer programs for children in families who are unable to afford these programs could result in a larger public health impact than enhancing existing practice within SDCs for only those children who can afford to attend. Given national data indicates there is a major opportunity gap among those that can access SDCs [32, 48, 63], with the majority of youth attending SDCs are White and from high-income households, providing access to this health enhancing environment to children from low-income neighborhoods could potentially address health inequities and disparities that have been widening over time.

Our secondary analyses, which focused on exposure to structured programming both during the school year and summer, showed attending structured programming at either time period provides health behavior benefits. These findings are consistent with the hypothesized mechanisms outlined in the Structured Days Hypothesis and demonstrate that environmental control via structure serves as an important modifier of child behaviors and leads to engagement in more beneficial behaviors (e.g., MVPA), and less engagement in unhealthy behaviors (e.g., screen time). Interestingly, for screen time, attending structure during both school and summer was associated with a sizable reduction in screen use compared to days where children did not attend structure. This suggests that additional structure has the ability, at least for screens, to minimize opportunities to use them (e.g., cell phones, tablets). Placing children in settings where screen use is not allowed could serve as a viable intervention strategy.

The strengths of the current study are the robust randomized design methodology, the large number of days of objectively measured behaviors for analysis, the detailed exposure measure collected in both the intervention and control condition, the focus on children from low-income households, and the practical approach to addressing unhealthy behaviors during the summer by providing access to existing community-operated summer programs. The limitations of the current study are the findings are restricted to a single geographical region in the southeastern United States, the lack of complete accelerometer data on all children randomized, and the parent self-report of screen time. Despite the restriction of generalizability, this region of the US has the greatest proportion of adverse health conditions and therefore, the findings, at minimum, generalize to this area of highest need. Second, although children who provided accelerometer data in the intervention and control group were virtually identical, there were some differences in demographic characteristics between children randomized to the intervention group who did and did not provide accelerometry data. This suggests some differential bias in our secondary outcome for the intervention group. To address this, we utilized these attributes in our imputation models as well as covaried for them in our models to account for their potential impact on the model derived estimated activity and sedentary behaviors. We used parent self-report of screen time, which may over-/under-estimate actual screen use. Finally, we are unable to determine whether “usual practice” in the current study represents “usual practice” across the entire US. The daily schedules of SDCs across the US may vary substantially and with this have differential impact on health behaviors.

In summary, our findings have broad implications for intervening during the summer to address unhealthy lifestyle behaviors (i.e., physical activity, screen use and time spent sedentary) and indicate leveraging existing community-operated programming – such as SDCs – and providing access to those who could not otherwise afford can lead to marked improvements in health behaviors linked to the risk of unhealthy weight gain during summer. Our findings can help inform policy decisions for providing financial support over the summer for access to existing community operated SDCs for youth from lower-income households.

## Supplementary Information

Supplementary Material 1

## Data Availability

Data will be made available immediately upon publication of the final scientific article specified in the grant application. Individuals requesting the data will be able to contact the PI or other members of the study team to receive instructions.
