# Supplementary material for: Impact of free summer day camp on physical activity behaviors and screentime of elementary-age children from low-income households: a randomized clinical trial
Source: Int J Behav Nutr Phys Act. 2025 Dec 29;22:159. doi: 10.1186/s12966-025-01852-2 (PMC12751614; doi:10.1186/s12966-025-01852-2)
Supplement: Supplementary file 1 — Supplementary Material 1 [file 12966_2025_1852_MOESM1_ESM.docx]

**Supplemental eTable 1.** Comparison of baseline child and household demographics between children with and without accelerometer data during school and during summer for the intervention (i.e., free summer day camp) and control (i.e., summer as usual) groups.

|  | **Control (Summer As Usual)** | | | | | | | | | | | | | | | | |  | **Intervention (Free Summer Day Camp)** | | | | | | | | | | | | | | | | |
| --- | --- | --- | --- | --- | --- | --- | --- | --- | --- | --- | --- | --- | --- | --- | --- | --- | --- | --- | --- | --- | --- | --- | --- | --- | --- | --- | --- | --- | --- | --- | --- | --- | --- | --- | --- |
|  | **SCHOOL** | | | | | | | |  | **SUMMER** | | | | | | |  |  | **SCHOOL** | | | | | | | |  | **SUMMER** | | | | | | |  |
|  | ACC | | |  | NO ACC | | | P-Value |  | ACC | | |  | NO ACC | | | P-Value |  | ACC | | |  | NO ACC | | | P-Value |  | ACC | | |  | NO ACC | | | P-Value |
| Sample Size | 136 |  |  |  | 112 |  |  |  |  | 161 |  |  |  | 73 |  |  |  |  | 159 |  |  |  | 101 |  |  |  |  | 189 |  |  |  | 64 |  |  |  |
| Child Characteristics |  |  |  |  |  |  |  |  |  |  |  |  |  |  |  |  |  |  |  |  |  |  |  |  |  |  |  |  |  |  |  |  |  |  |  |
| Sex (Females, %) | 49% |  |  |  | 55% |  |  | 0.376 |  | 49% |  |  |  | 57% |  |  | 0.298 |  | 42% |  |  |  | 52% |  |  | 0.164 |  | 47% |  |  |  | 42% |  |  | 0.516 |
| Age (years, mean, ±SD) | 8.2 | ±1.6 |  |  | 8.2 | ±1.1 |  | 0.835 |  | 8.6 | ±1.6 |  |  | 8.0 | ±1.4 |  | 0.020 |  | 8.2 | ±1.6 |  |  | 7.9 | ±1.5 |  | 0.196 |  | 8.4 | ±1.5 |  |  | 8.6 | ±1.8 |  | 0.532 |
| Self-Identified Race/Ethnicity, % |  |  |  |  |  |  |  | 0.103 |  |  |  |  |  |  |  |  | 0.075 |  |  |  |  |  |  |  |  | 0.010 |  |  |  |  |  |  |  |  | 0.008 |
| Hispanic | 8% |  |  |  | 2% |  |  |  |  | 6% |  |  |  | 7% |  |  |  |  | 11% |  |  |  | 12% |  |  |  |  | 10% |  |  |  | 16% |  |  |  |
| Black | 42% |  |  |  | 59% |  |  |  |  | 45% |  |  |  | 52% |  |  |  |  | 43% |  |  |  | 65% |  |  |  |  | 44% |  |  |  | 64% |  |  |  |
| White | 37% |  |  |  | 27% |  |  |  |  | 39% |  |  |  | 20% |  |  |  |  | 39% |  |  |  | 13% |  |  |  |  | 36% |  |  |  | 16% |  |  |  |
| Other Race | 3% |  |  |  | 3% |  |  |  |  | 2% |  |  |  | 6% |  |  |  |  | 6% |  |  |  | 9% |  |  |  |  | 8% |  |  |  | 2% |  |  |  |
| Multiple Races | 10% |  |  |  | 9% |  |  |  |  | 8% |  |  |  | 15% |  |  |  |  | 1% |  |  |  | 1% |  |  |  |  | 1% |  |  |  | 2% |  |  |  |
| Anthropometrics (baseline) |  |  |  |  |  |  |  |  |  |  |  |  |  |  |  |  |  |  |  |  |  |  |  |  |  |  |  |  |  |  |  |  |  |  |  |
| BMI Z-Score (Median, IQR) | 0.74 | (-0.1, | 1.6) |  | 0.73 | (0.1, | 1.6) | 0.906 |  | 0.76 | (-0.2, | 1.5) |  | 1.05 | (0.2, | 1.8) | 0.308 |  | 0.75 | (0.0, | 1.8) |  | 0.54 | (-0.0, | 1.4) | 0.337 |  | 0.79 | (-0.0, | 1.8) |  | 0.37 | (-0.3, | 1.6) | 0.384 |
| BMI classifications (%) |  |  |  |  |  |  |  | 0.420 |  |  |  |  |  |  |  |  | 0.296 |  |  |  |  |  |  |  |  | 0.757 |  |  |  |  |  |  |  |  | 0.583 |
| Normal Weight | 60% |  |  |  | 65% |  |  |  |  | 63% |  |  |  | 56% |  |  |  |  | 63% |  |  |  | 70% |  |  |  |  | 64% |  |  |  | 66% |  |  |  |
| Overweight | 22% |  |  |  | 18% |  |  |  |  | 18% |  |  |  | 27% |  |  |  |  | 16% |  |  |  | 12% |  |  |  |  | 14% |  |  |  | 16% |  |  |  |
| Obese | 18% |  |  |  | 18% |  |  |  |  | 18% |  |  |  | 18% |  |  |  |  | 21% |  |  |  | 19% |  |  |  |  | 22% |  |  |  | 18% |  |  |  |
| Household/Family (%) |  |  |  |  |  |  |  |  |  |  |  |  |  |  |  |  |  |  |  |  |  |  |  |  |  |  |  |  |  |  |  |  |  |  |  |
| Food Insecure | 31% |  |  |  | 38% |  |  | 0.285 |  | 37% |  |  |  | 24% |  |  | 0.073 |  | 27% |  |  |  | 28% |  |  | 0.913 |  | 25% |  |  |  | 35% |  |  | 0.167 |
| At or Below 200% Federal Poverty Level | 62% |  |  |  | 63% |  |  | 0.927 |  | 60% |  |  |  | 69% |  |  | 0.279 |  | 65% |  |  |  | 78% |  |  | 0.050 |  | 62% |  |  |  | 85% |  |  | 0.002 |
| Services Received (%) |  |  |  |  |  |  |  |  |  |  |  |  |  |  |  |  |  |  |  |  |  |  |  |  |  |  |  |  |  |  |  |  |  |  |  |
| Welfare, Temporary Assistance for Needy Families (TANF),  or Temporary Cash Assistance (TCA) | 20% |  |  |  | 17% |  |  | 0.571 |  | 22% |  |  |  | 11% |  |  | 0.076 |  | 16% |  |  |  | 11% |  |  | 0.331 |  | 13% |  |  |  | 17% |  |  | 0.488 |
| Children's Health Insurance Program (CHIP), Medicaid | 52% |  |  |  | 50% |  |  | 0.805 |  | 49% |  |  |  | 58% |  |  | 0.227 |  | 51% |  |  |  | 60% |  |  | 0.250 |  | 49% |  |  |  | 66% |  |  | 0.031 |
| Supplemental Nutrition Assistance Program (SNAP) | 17% |  |  |  | 44% |  |  | 0.000 |  | 25% |  |  |  | 29% |  |  | 0.522 |  | 26% |  |  |  | 29% |  |  | 0.666 |  | 25% |  |  |  | 32% |  |  | 0.321 |
| Women, Infants and Children (WIC) | 9% |  |  |  | 12% |  |  | 0.472 |  | 8% |  |  |  | 16% |  |  | 0.062 |  | 6% |  |  |  | 20% |  |  | 0.003 |  | 12% |  |  |  | 6% |  |  | 0.190 |
| Parent Education (%) |  |  |  |  |  |  |  | 0.091 |  |  |  |  |  |  |  |  | 0.945 |  |  |  |  |  |  |  |  | 0.407 |  |  |  |  |  |  |  |  | 0.123 |
| Some college or less | 41% |  |  |  | 53% |  |  |  |  | 44% |  |  |  | 46% |  |  |  |  | 51% |  |  |  | 61% |  |  |  |  | 50% |  |  |  | 65% |  |  |  |
| 2 to 4yr degree | 35% |  |  |  | 35% |  |  |  |  | 36% |  |  |  | 33% |  |  |  |  | 34% |  |  |  | 28% |  |  |  |  | 36% |  |  |  | 24% |  |  |  |
| Graduate degree | 24% |  |  |  | 12% |  |  |  |  | 21% |  |  |  | 20% |  |  |  |  | 14% |  |  |  | 12% |  |  |  |  | 15% |  |  |  | 11% |  |  |  |
| Family Income (%) |  |  |  |  |  |  |  | 0.931 |  |  |  |  |  |  |  |  | 0.229 |  |  |  |  |  |  |  |  | 0.154 |  |  |  |  |  |  |  |  | 0.000 |
| $30k or less | 39% |  |  |  | 42% |  |  |  |  | 38% |  |  |  | 44% |  |  |  |  | 49% |  |  |  | 60% |  |  |  |  | 49% |  |  |  | 58% |  |  |  |
| $30k to $60k | 32% |  |  |  | 29% |  |  |  |  | 29% |  |  |  | 35% |  |  |  |  | 23% |  |  |  | 26% |  |  |  |  | 21% |  |  |  | 36% |  |  |  |
| $60k or more | 29% |  |  |  | 29% |  |  |  |  | 33% |  |  |  | 20% |  |  |  |  | 28% |  |  |  | 16% |  |  |  |  | 31% |  |  |  | 5% |  |  |  |

**Supplemental eTable 2.** Daily summer day camp schedules for summer 2021, 2022, and 2023. Grey indicates physical activity opportunities.

| **Year** | **Time** | **Activity** |  | **Field Trip examples across all years** |
| --- | --- | --- | --- | --- |
| 2021 | 7:30-8:30am | AM drop off- Auditorium |  | Movie Theater |
|  | 8:30-9:00am | Indoor games or Enrichment Activity |  | Splash Pad |
|  | 9:00-9:45am | Outside/Gym |  | Bowling |
|  | 10:30-11:30am | Outside/ Playground |  | Zoo |
|  | 11:25-11:50am | Lunch |  | Children’s Museum |
|  | 11:50-12:45pm | Indoor games or Enrichment Activity |  |  |
|  | 12:45-1:30pm | Outside/Gym |  |  |
|  | 1:30-2:15pm | Indoor games or Enrichment Activity |  |  |
|  | 2:15-3:00pm | Indoor games or Enrichment Activity |  |  |
|  | 3:00-3:30pm | Snack |  |  |
|  | 3:30-4:15pm | Outside/Gym |  |  |
|  | 4:15-5:00pm | Indoor games or Enrichment Activity |  |  |
|  | 5:00-6:00pm | Auditorium for pick up |  |  |
|  |  |  |  |  |
| 2022 | 7:30-8:30am | AM drop off- Auditorium |  |  |
|  | 8:30-9:00am | Breakfast |  |  |
|  | 9:00-10:00am | Outside/ Playground |  |  |
|  | 10:00-11:00am | Indoor games or Enrichment Activity |  |  |
|  | 11:00-12:00pm | Outside/Gym |  |  |
|  | 12:00-1:00pm | Lunch |  |  |
|  | 1:00-2:00pm | Indoor games or Enrichment Activity |  |  |
|  | 2:00-3:00pm | Indoor games or Enrichment Activity |  |  |
|  | 4:00-5:00pm | Outside/Gym |  |  |
|  | 5:00-6:00pm | Auditorium for pick up |  |  |
|  |  |  |  |  |
| 2023 | 7:30-8:15am | AM drop off- Auditorium |  |  |
|  | 8:30-9:00am | Breakfast |  |  |
|  | 9:00-10:30am | Outside/Gym |  |  |
|  | 10:30-11:45am | Indoor games or Enrichment Activity |  |  |
|  | 11:45-12:30pm | Lunch |  |  |
|  | 12:30-2:30pm | Enrichment Activity |  |  |
|  | 2:45-3:15pm | Snack |  |  |
|  | 3:30-4:30pm | Outside/Gym |  |  |
|  | 4:30-5:15pm | Indoor games or Activity |  |  |
|  | 5:15-6:00pm | Auditorium for pick up |  |  |
